# Pain, PSA flare, and bone scan response in a patient with metastatic castration-resistant prostate cancer treated with radium-223, a case report

**DOI:** 10.1186/s12885-015-1390-y

**Published:** 2015-05-07

**Authors:** Megan A McNamara, Daniel J George

**Affiliations:** 1 Division of Medical Oncology, Duke University Medical Center, Durham, NC USA; 2 Duke University Medical Center, 1 Trent Drive, Morris Building Rm #25169, Box 3841, Durham, NC 27710 USA; 3 Duke University Medical Center, 10 Bryan Searle Drive, 471 Seeley Mudd Bldg, Box 102002, Durham, NC 27710 USA

**Keywords:** Radium-223, Flare, Bone scan, Response

## Abstract

**Background:**

Radium-223 has been shown to improve overall survival in men with metastatic castration-resistant prostate cancer with symptomatic bone metastases. The bone scan response to radium-223 has only been described in one single center trial of 14 patients, none of whom achieved the outstanding bone scan response presented in the current case.

**Case presentation:**

In this case report, we describe a 75 year-old white man with extensively pre-treated metastatic castration-resistant prostate cancer and symptomatic bone metastases who experienced a flare in pain and prostate-specific antigen, followed by dramatic clinical (pain), biochemical (prostate-specific antigen), and imaging (bone scan) response.

**Conclusion:**

The flare phenomena and bone scan response we observed have not previously been described with radium-223. This case suggests that the degree and duration of bone scan response may be predictive of overall survival benefit.

## Background

Prostate cancer is the most common non-cutaneous malignancy in US men and is the second leading cause of cancer-related mortality [1]. In 2014, it is estimated that 29,480 men died from metastatic castration-resistant prostate cancer (mCRPC), the terminal form of the disease. Among patients with mCRPC, bone is the most frequent site of metastatic disease. These metastases are characterized by high uptake of technetium phosphate on bone scan and represent calcium deposition in areas of osteoblastic-driven bone turnover. Bone metastases cause significant morbidity, including pain requiring palliative radiation, pathologic fractures, spinal cord compression, and orthopedic surgery, otherwise referred to as symptomatic skeletal events (SSE) [2-4], and are independently associated with increased mortality in patients with mCRPC [5].

Radium-223 dichloride (Radium-223, Xofigo, previously alpharadin) is a first-in-class alpha-emitting radionuclide, approved for the treatment of men with mCRPC with symptomatic bone metastases and no known visceral metastases. It acts as a calcium-mimetic and is preferentially taken up into areas of high bone turnover, such as those surrounding bone metastases [6,7]. Once radium-223 reaches bone, it emits alpha-particle radiation, which induces double stranded breaks in DNA, causing a local cytotoxic effect [6,8]. Importantly, because alpha particles have a very short range (<100 μm), there is limited damage to surrounding normal tissues, including bone marrow [7,9]. In early drug testing, radium-223 was given safely with repeated dosing every four weeks [10-14].

A randomized phase III clinical trial (ALSYMPCA, alpharadin in symptomatic prostate cancer) demonstrated that treatment with radium-223 every four weeks for up to six months significantly prolonged the survival of men with mCRPC with bone metastases, compared to placebo [4], leading to FDA approval in this setting in May 2013. This survival benefit distinguishes radium-223 from other therapies, including local radiation, radioisotopes strontium-89 and samarium-153, zoledronic acid, and denosumab, which have been shown to decrease pain from bone metastases but have not shown an improvement in overall survival [15-17]. The ALSYMPCA trial also demonstrated that treatment with radium-223 prolonged the time to first symptomatic skeletal event, improved alkaline phosphatase, enhanced quality of life, and was well tolerated. A prostate-specific antigen (PSA) response was observed in a minority of patients [4].

Interestingly, the bone scan response to treatment with radium-223 was not described in the ALSYMPCA trial. In fact, there is currently no validated method to quantify decreased tumor burden on bone scan, and to our knowledge, the bone scan response to radium-223 has only been described in one single center trial of 14 patients, in which bone scans were obtained at baseline and one month after the 6th dose of radium-223. In this study, bone scans were manually reviewed by two radiologists, who counted the number of new metastatic lesions and scored the bone scans according to the Soloway extent of disease classification. After treatment with radium-223, most patients in this study (10 of 12 evaluable) demonstrated decreased radiotracer uptake in existing lesions. However, new areas of uptake, consistent with new osseous metastatic lesions, developed in 11 of 12 of these patients [18].

The following case report presents a 75 year-old otherwise-healthy white male, who presented with metastatic prostate cancer with a single, asymptomatic osseous metastasis. He was extensively treated with androgen deprivation therapy, combined androgen blockade, immunotherapy with sipuleucel-T, docetaxel chemotherapy, enzalutamide, and palliative external beam radiation, with varying degree and duration of response. He eventually developed mCRPC with symptomatic, widespread osseous metastases and was treated with radium-223, with an initial flare in pain and PSA, followed by dramatic and durable improvement in pain, alkaline phosphatase, and bone scan.

## Case presentation

**Figure 1:**
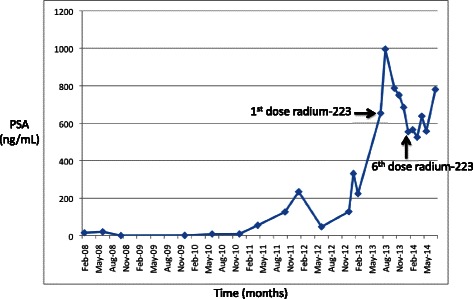
PSA trend and response to radium-223. The trend in the patient’s PSA over the course of his disease is shown, and the PSA response to radium-223 is highlighted. PSA flared after the first dose of radium-223, peaking at 996 ng/mL 3 weeks after the initiation of therapy. PSA then steadily improved and nadired at 554 ng/mL about 1 month after the 6th dose of radium-223. After completion of radium-223, PSA remained stable at about 600 ng/mL for about 7 months before increasing again.

**Figure 2:**
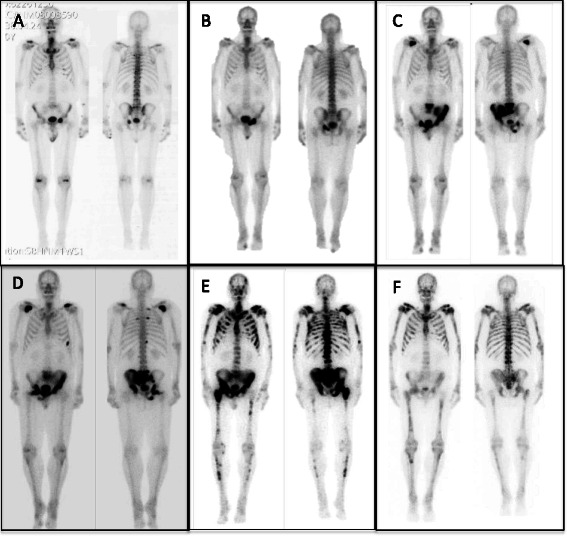
Radionuclide bone scan trend and response to radium-223. **A**. June 2008, metastatic prostate cancer diagnosis. **B**. May 2010, disease progression on combined androgen blockade. **C**. May 2011, disease progression following sipuleucel-T immunotherapy. **D**. December 2011, disease progression just prior to docetaxel chemotherapy. **E**. June 2013, widespread disease progression associated with severe diffuse bone pain on enzalutamide (“pre-radium-223”). **F**. February 2014, dramatic bone scan response two months after completing six treatments of radium-223 (“post-radium-223”).

Our patient first presented in February 2008 with lower urinary tract symptoms and was found to have an elevated PSA at 14.8 ng/mL (Figure 1). Subsequent transrectal ultrasound-guided biopsy of the prostate showed high-grade prostate adenocarcinoma, with Gleason Score 4 + 5 = 9 and 4 + 4 = 8, in 10 of 13 cores, in all areas of the prostate. Because of the high-grade disease, staging studies were performed. Bone scan showed increased uptake in the left pubic ramus (Figure 2A). CT scan of the abdomen and pelvis did not show lymphadenopathy or intraabdominal disease but did demonstrate blastic activity in the left superior pubic ramus, corresponding to the region of increased uptake seen on bone scan. Overall, this presentation was felt to be consistent with metastatic prostate cancer with a single, asymptomatic, bone metastasis.

In June 2008, he was initiated on androgen deprivation therapy (ADT), which he has remained on, with or without additional therapies, since then. Please refer to Table 1 for full details of his treatment course prior to radium-223. Briefly, from June 2010 to June 2013, he was treated with a DHEAS inhibitor on a Phase I clinical trial, sipuleucel-T immunotherapy, a CYP17 inhibitor plus prednisone on a Phase III clinical trial, 10 cycles of docetaxel chemotherapy plus prednisone, and enzalutamide, as well as two courses of palliative radiation to painful osseous metastases. He had transient improvement in his mCRPC in response to many of these therapies, but overall his course was characterized by disease progression, with rising PSA, increasing alkaline phosphatase, and growing osseous metastases.

**Table 1:** **Timeline**

Date	PSA (ng/mL)	Imaging	Bone pain level	Therapy
February 2008	14.8	Single bone metastasis in left pubic ramus	None	
June 2008	20.1		None	ADT initiated
October 2008	0.2		None	
June 2010	8.0	Progression: increase in the extent of abnormal radiotracer activity within the left acetabulum and left superior pubic ramus	None	Phase I study of BN83495 (DHEAS inhibitor)
July 2010	13		Moderate	Taken off study for PSA progression; Palliative radiation to pelvic bone metastasis for left hip pain
December 2010-January 2010			None	Sipuleucel-T immunotherapy
February 2011		Progression: new osseous metastases; no visceral metastases	None	
April 2011	54.8		None	
May 2011		Progression: new osseous metastases; no visceral metastases	None	
June 2011			None	Phase III study of CYP17 inhibitor plus prednisone (randomized to CYP17 inhibitor arm)
October 2011	126.4	Progression: new osseous metastases; no visceral metastases	Moderate	Taken off study for symptomatic disease progression
January 2012-July 2012	234 (January) 46.95 (July)		Moderate → mild	10 cycles of docetaxel chemotherapy plus prednisone
August 2012 and October 2012		Stable osseous metastatic disease; no visceral metastases	Mild	
December 2012	128		Moderate	Palliative radiation to a sacral osseous metastasis for low back pain
January 2013	331		Mild	Enzalutamide initiated
February 2013	224		Mild	
July 2013	653	Progression of osseous metastatic disease; no visceral metastases	Severe diffuse bone pain	
August 2013	996		Pain flare for 10 days after 1st dose of radium-223	1st dose of radium-223
October 2013			Pain significantly improved, discontinued opioids	
November 2013			Mild pain controlled with NSAIDs	Symptomatic anemia, requiring transfusion. Has remained transfusion-dependent since.
December 2013			Mild pain controlled with NSAIDs	6th dose radium-223
January 2014	554		Mild pain controlled with NSAIDs	
February 2014		Dramatic bone scan response to radium-223 with significant decrease in activity of all skeletal metastases	Mild pain controlled with NSAIDs	
January-July 2014	Stable at about 600		Mild pain controlled with NSAIDs	
July 2014	780	Out-of-field disease progression: new diffuse osseous metastases in the skull, no change in previously existing metastatic disease	Headache; Bone pain stable (mild)	Steroids and stereotactic radiation to skull base mass

**Figure 3:**
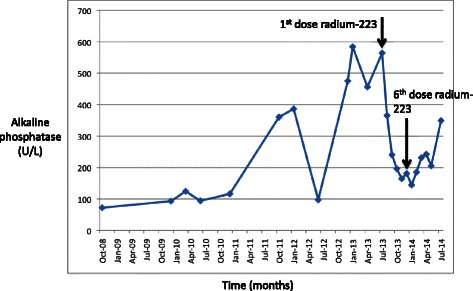
Alkaline phosphatase trend and response to radium-223. The trend in the patient’s alkaline phosphatase over the course of his disease is shown, and the alkaline phosphatase response to radium-223 is highlighted. Alkaline phosphatase was 564 U/L at the time of initiation of radium-223 and steadily improved throughout the course of radium-223. Alkaline phosphatase nadired at 144 U/L about 1 month after the 6th dose of radium-223 and then trended up again.

By July 2013, PSA had reached 653 ng/mL (Figure 1) and alkaline phosphatase was 564 U/L (Figure 3). He had also developed severe diffuse bone pain. Re-staging scans showed disease progression with increase in the size of diffuse bone metastases, some of which were now confluent (Figure 2E). There was still no evidence of visceral metastatic disease.

On August 1, 2013, he received his first dose of radium-223 50 kBq/kg. Enzalutamide (and leuprolide) were continued. Following the first dose of radium-223, he experienced a flare in his bone pain, which he described as “severe, total body pain” that caused him to be bedbound for 10 days. A concurrent flare in PSA was also observed, with PSA peaking at 996 ng/mL three weeks after the first dose of radium-223 (Figure 1). After the initial flare, bone pain and PSA steadily improved. He was able to discontinue opioids within eight weeks of start of therapy. After his fifth dose of radium-223, he developed symptomatic anemia with hemoglobin 7.5 g/dL. He was transfused two units of packed red blood cells and has remained red blood cell transfusion-dependent since then, requiring transfusions every two to four weeks. He completed his sixth dose of radium-223 in December 2013. PSA nadired at 554 ng/mL in January 2014 (Figure 1). Alkaline phosphatase dropped steadily throughout the course of radium-223 and nadired at 144 U/L in January 2014 (Figure 3). Re-staging bone scan in February 2014 showed a dramatic response to radium-223 with significant decrease in activity of all skeletal metastases (Figure 2F).

He had a sustained excellent response to radium-223, with continued pain relief, requiring only as-needed NSAIDs, and stable PSA of about 600 ng/mL until July 2014 (Figure 1), when he reported new right temporal headache and diplopia. Brain MRI at that time showed interval development of new diffuse osseous metastatic disease in the skull, not present at the time of radium, with intracranial and intraorbital mass extension, exerting mass effect on the lateral rectus and optic nerve. Bone scan confirmed the MRI findings and showed no change in the appearance of his previously existing metastatic disease. PSA had increased to 780 ng/mL (Figure 1). He was started on steroids and underwent stereotactic radio-surgery (SRS) to the skull base mass, with resolution of his headaches and diplopia.

## Conclusions

There are four unique features of this case that have not been well described in the literature. First, the pain flare phenomenon that our patient experienced has only been reported in a Phase I trial of radium-223, in which seven of twenty-five patients had a “flare” in pain during the first week of radium-223 treatment [11]. In our case, the pain flare involved multiple sites and resulted in a transient decrease in performance status that lasted 10 days. Subsequent radium-223 doses were associated with much less or no pain flare.

Second, the PSA flare phenomenon that we observed has not been described following treatment with radium-223. In our clinical experience, other forms of radiotherapy have also been associated with a transient increase in PSA. We hypothesize that the observed PSA flare after radium-223 treatment is due to PSA release from tumor cell lysis. Based on this mechanism of action and on our experience with this patient, PSA flare may correspond to a high degree of tumor cell kill and may be associated with more complete and more durable response to radium-223.

Third, we are aware of one other case report that includes a dramatic bone scan response to radium-223, similar to what we observed in our patient [19]. As mentioned previously, in the only study to date that has described the bone scan response to radium-223, the majority of patients experienced decreased radiotracer uptake in existing bone lesions with simultaneous development of new osseous metastatic lesions. None of the patients in that study demonstrated the excellent bone scan response that we saw in our patient. Additionally, that study included only a single post-radium-223 bone scan, so the duration of response in those patients is unknown [18]. This is in contrast to our patient, who maintained his outstanding response for seven months after completion of radium-223.

**Figure 4:**
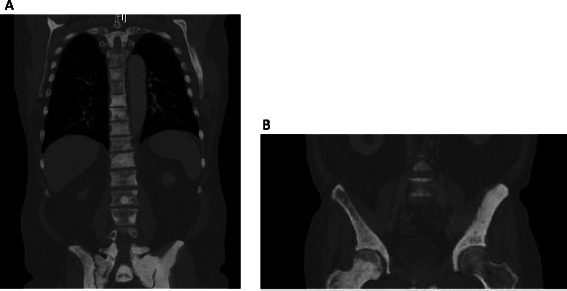
Diffuse sclerosis, resulting from the patient’s high osseous tumor burden and associated high radium-223 uptake. Diffuse osseous sclerosis on CT imaging of the spine **(A)**, bilateral ilia **(B)**, and bilateral femoral heads **(B)** is shown. This sclerosis occurred because of the patient’s high volume of bony metastatic disease and consequential high radium-223 uptake. We believe that this combination of high osseous tumor burden and high radium-223 uptake, resulting in diffuse sclerosis, contributed to our patient’s transfusion dependence, which has persisted in the absence of continued radium-223 therapy or systemic disease progression.

Finally, anemia is a known poor prognostic factor in mCRPC and a known side effect of radium-223. Red blood cell transfusions are required in about twice as many patients receiving radium-223 with prior docetaxel use, compared to patients with no prior docetaxel use [20]. However, continued transfusion dependence in the absence of continued radium-223 therapy or systemic disease progression, like we observed in our patient, has not been described. We attribute our patient’s transfusion dependence to his high tumor burden and associated high radium uptake, which resulted in diffuse sclerosis on CT of his pelvis and other osseous sites (Figure 4). Prior chemotherapy and radiation therapy likely also contributed to the development of transfusion-dependent anemia in this patient. Interestingly, this has not affected his survival (>1 year since becoming transfusion dependent), likely because it is not from disease progression.

We conclude that treatment of men with mCRPC with radium-223 can cause a flare in pain and PSA after the first dose of therapy and can also achieve an outstanding bone scan response. Additionally, there is a risk of red blood cell transfusion dependence in extensively pre-treated patients with high burden of osseous metastases, which should not necessarily be viewed as a poor prognostic sign. Knowledge of the potential for a flare in pain and PSA has implications for treatment, since it is important to realize that a flare does not necessarily indicate lack of response to radium-223 and may in fact be predictive of an excellent overall response, as demonstrated in our patient. Based on our experience with this patient, we would recommend counseling patients beginning radium-223 about the possibility of pain and PSA flare. In the event that a pain flare occurs, we suggest pain management with as needed acetaminophen and oxycodone.

The duration of objective imaging response in this case and the subsequent progression in new sites of disease suggests a change in the tumor environment of the treated disease sites that is more durable, compared to patients who develop disease progression in existing disease sites after treatment with radium-223. Furthermore, based on our experience with this patient, who, at the time of submission, is still alive 16 months after the first dose of radium-223, an excellent bone scan response may be predictive of a better overall survival benefit and should be evaluated in larger series. Transfusion dependence could be avoided by treating similar patients with radium-223 earlier in their disease course. Further studies are needed to better understand the bone scan response to radium-223.

## Consent

Written informed consent was obtained from the patient for publication of this Case report and any accompanying images. A copy of the written consent is available for review by the Editor of this journal.

Per our institutional policy, we also obtained IRB approval for this Case (IRB number Pro00059303).

## Abbreviations

mCRPC: metastatic castration-resistant prostate cancer
SSE: Symptomatic skeletal event
ALSYMPCA: Alpharadin in symptomatic prostate cancer
PSA: Prostate-specific antigen
ADT: Androgen deprivation therapy
